# Beyond the Mammalian Heart: Fish and Amphibians as a Model for Cardiac Repair and Regeneration

**DOI:** 10.3390/jdb4010001

**Published:** 2015-12-23

**Authors:** Kyle Jewhurst, Kelly A. McLaughlin

**Affiliations:** Department of Biology, Tufts University, Medford, MA 02155, USA; Kyle.Jewhurst@tufts.edu

**Keywords:** heart, regeneration, apoptosis, necrosis, myocardium, epicardium, fibrosis, migration, proliferation, dedifferentiation, transdifferentiation, retinoic acid, TGF-β, FGF

## Abstract

The epidemic of heart disease, the leading cause of death worldwide, is made worse by the fact that the adult mammalian heart is especially poor at repair. Damage to the mammal heart—such as that caused by myocardial infarction—leads to scarring, resulting in cardiac dysfunction and heart failure. In contrast, the hearts of fish and urodele amphibians are capable of complete regeneration of cardiac tissue from multiple types of damage, with full restoration of functionality. In the last decades, research has revealed a wealth of information on how these animals are able to perform this remarkable feat, and non-mammalian models of heart repair have become a burgeoning new source of data on the morphological, cellular, and molecular processes necessary to heal cardiac damage. In this review we present the major findings from recent research on the underlying mechanisms of fish and amphibian heart regeneration. We also discuss the tools and techniques that have been developed to answer these important questions.

## 1. Introduction

The human heart is an organ of both vital importance and startling fragility. Heart disease is the leading cause of death worldwide, responsible for one in every four deaths [1]. In the United States this figure is even higher, with one-third of all deaths attributable to heart disease. In addition, 8 million Americans have suffered a myocardial infarction at some point in their lives, and one third of adult Americans are afflicted with hypertension, which can have detrimental effects on heart physiology and function [2]. In short, heart disease is an epidemic unlike any other. Thus, a greater understanding of the causes of heart disease, and treatment for its most damaging effects, is of the greatest necessity.

The major difficulty in developing treatments for cardiac tissue damage is that the adult mammalian heart is particularly poor at repairing post-injury. Infarction or physical damage to mammal hearts often results in permanent scarring, leading to cardiac remodeling and heart dysfunction, and ultimately contributing to progressive heart failure [3]. Research into mammalian heart repair has generally followed two strategies—stem cell/cardiac progenitor-based therapies, and endogenous pathways of repair [4]. Once thought to be distinct processes, these two approaches may in fact be more related than previously believed. Some studies indicate that stem cell-based strategies work by acting indirectly on existing heart cells and stimulating endogenous repair processes, rather than any direct contribution to restoring tissue by the stem cells themselves [4,5]. An understanding of the inherent repair capabilities of the heart, therefore, seems to be the most comprehensive approach towards developing novel methods of treatment.

For decades, the mammalian heart was believed to be a post-mitotic organ, and therefore incapable of healing itself after damage. However, more recent research has shown that low levels of mitosis occur in mature cardiomyocytes, and new myocytes are routinely generated from pre-existing ones as part of the heart’s natural maintenance of homeostasis [6]. Increased proliferation of myocytes adjacent to myocardial infarcts has also been seen in human patients, though this process is not sufficient to fully repair damage [7]. Cardiac regeneration—that is, restoration of muscle with limited to no scarring—has even been observed in neonatal mice under a wide variety of damage models [8,9,10,11,12], though the extent of regeneration these animals are capable of has been disputed [13]. Research into the inherent reparative and regenerative capacity of mammalian hearts thus shows promise for developing strategies to combat and repair heart damage in humans. However, since mammal embryos are difficult to manipulate and closely observe during repair, and adult mammal hearts are incapable of regenerating, the potential of mammalian models remains limited for understanding how heart tissue can be regenerated.

In recent years, therefore, the scientific community has begun to take a greater interest in non-mammalian hearts as a model for heart repair. In particular, the so-called “lower vertebrates”, amphibians and fish, have proven themselves to be highly tractable model systems in this field of study. The fact that these animals can regenerate cardiac tissue has been known for some time; forty years ago, Oberpriller and Oberpriller discovered that damage to the hearts of adult newts caused cardiomyocytes near the wound site to proliferate, a sign of regenerative capacity [14]. That same year, Becker and colleagues showed that the salamander heart could fully restore itself after injury, both functionally and histologically [15]. In the following decades, further research was conducted on heart regeneration in urodele amphibians, most commonly the newt *Notophthalmus viridescens* [16,17,18], but also the axolotl *Amblystoma mexicanum* [19,20] and the salamander *Triturus viridescens* [15]. Many of the gross morphological and cellular events of cardiac regeneration were first characterized in amphibian systems, and they remain important as models for studying regenerative processes.

It was not until 2002 that a species outside of amphibians was found to be capable of cardiac regeneration in adult organisms—the zebrafish, *Danio rerio*. Poss and colleagues demonstrated that zebrafish hearts are capable of fully regenerating missing cardiac tissue, with no sign of scarring and full restoration of functionality, within 60 days after injury [21]. Since that discovery, *D. rerio* has become, by far, the most utilized and well-characterized model organism for studying heart regeneration. With a wealth of molecular tools and genomic data available for the species, profiling gene expression, creating transgenic organisms, and mutagenesis are made significantly easier than in less well-characterized model systems [21]. The morphological and cellular processes during zebrafish heart regeneration show a great deal of conservation with those same processes in amphibians, but the tools available to study zebrafish have made it possible to delve even further into the underlying molecular pathways. While zebrafish have been the primary model for cardiac regeneration, other species of teleost fish are capable of it as well, allowing for opportunities to study the evolutionary conservation of regenerative processes. Fish species in which heart regeneration has been studied include *Danio aequippinatus*, a close relative to the zebrafish [22], the goldfish *Carassius auratus*, a more distant relative of the *Danio* genus [23], and *Polypterus senegalus*, a member of the most basally branching group of ray-finned fishes [24]. However, the regenerative response is not present in all teleosts; at least one species, the medaka *Oryzias latipes*, scars instead of regenerating after cardiac trauma [25].

In the past decade, therefore, the field of cardiac regeneration has expanded rapidly. A wealth of research has been conducted in both amphibian and fish model systems, across numerous species, into how these animals are capable of restoring damaged or missing heart tissue. In this review, we discuss the major findings on heart regeneration in non-mammalian species, as well as the tools that have been developed to study this process.

## 2. Techniques for Studying Heart Regeneration and Repair

### 2.1. Mechanical Manipulation

As our knowledge of the processes of cardiac regeneration has advanced, so too have the tools we use to study it. The first developed, and by far most common, method of damaging hearts to study their repair is injury via mechanical manipulation. This broad category of techniques includes amputation of the ventricular apex [21], resection of the ventricular myocardium (away from the apex) [26], resection of the atrium [17], and mechanical squeezing or crushing [27].

Ventricular apical amputation is one of the most widely-used methods of injuring hearts, likely due to its highly visible phenotype and relatively easy access to the ventricular apex. The body of the animal and the pericardium are opened up to expose the heart. A pair of scissors is used to cut off the apex; in fish, approximately 20%–30% of the ventricle is removed [21,28], while in amphibians 10%–15% is standard [14,20]. In fish, the wound clots in less than a minute, and the animal is left to heal the incision in its abdomen [21]. In amphibians, sutures are required in the abdominal wall, and the animal is left at cold temperatures overnight to recover [14,20]. In all species, the survival rate of this procedure is 80%–90%, but amputating an excessive amount of the ventricle can greatly reduce this number [21]. Other variants of mechanical damage follow a similar procedure, with the major differences being where on the heart the incision takes place, or with what tools the damage is induced. These alternate methods may be used to target a different region of the heart, such as atrial resection [17], or to improve reproducibility and survivability, such as (non-apical) ventricular resection [26]. Because surgery through the abdominal wall may have its own effects on the repair response, sham-operated controls—consisting of an incision to access the heart, but no damage to the heart itself—are always necessary with this technique.

Although mechanical damage does not replicate any human heart disease in particular, it may be most applicable to the study of infarcts, where large regions of myocardial tissue undergo necrosis. However, the similarities are limited. In an infarct in the human heart, cellular debris remains at the wound site post-injury, and it must be removed before repair can begin; in a resected heart, no tissue is left behind, leaving one less step to be required before repair can take place [29]. Differences like these make it difficult to directly apply results from resection experiments into a human heart disease context. To this end, other damage models that better recapitulate certain aspects of heart disease have also been developed.

### 2.2. Cryoinjury

The cryoinjury model was developed to better mimic natural conditions of heart disease, specifically those found after myocardial infarction (MI). Unlike resection, which involves wholesale removal of tissue from the heart, cryoinjury induces tissue damage *in situ*, much like what would occur from ischemia during MI. Following cryoinjury, 25% of the ventricle undergoes necrosis or apoptosis, on a similar scale to MI-induced ischemia, after which fibrosis occurs much as it does in the mammalian heart. However, unlike in mammalian MI, regeneration follows in fish and amphibians, with the fibrotic tissue eventually replaced by functional myocardium [29]. Originally developed for mammalian studies, the cryoinjury model has been used for decades in studying repair of infarct-like wounding in mice and rats [30,31,32]. Since its adaptation for fish, cryoinjury has been extensively used in zebrafish models to study cardiac regeneration [29,33,34,35,36,37].

Cryoinjury results from application of extreme cold to living tissue. The change in temperature disrupts proteins and forms ice crystals that damage the plasma membrane, resulting in apoptosis and necrosis. Cryoinjury is commonly performed with a copper or stainless steel filament (<1 mm diameter) cooled by liquid nitrogen, known as the cryoprobe [29,34]. An alternate protocol uses a small (2 mm diameter) cone of dry ice instead [35]. After the animal is anesthetized, an incision is made in the body wall and pericardium to provide access to the heart. The cryoprobe is applied to the ventricle for a few seconds—the exact time varying by protocol—after which it is removed and the animal is left to heal naturally [29,34,35]. Mortality is greatly reduced compared to ventricular resection, with 5% or less subject death reported [35,36]. As with resection, sham-operated controls are necessary to account for the effects of opening the abdominal wall.

An interesting variation on this method—cautery, application of extreme heat—has been used in two heart regeneration studies, one in the giant danio (*D. aequippinatus*) and one in the goldfish (*Carassius auratus*). The procedure is performed in much the same way as cryoinjury, only using a heated filament; it has the same advantages as cryoinjury as well, leaving behind a region of necrotic tissue akin to that found in an infarct [22,23].

### 2.3. Inducible Transgenics

One of the major advantages of working with fish and amphibians over mammals is the relative ease of producing transgenic organisms. Because fish and amphibian embryos develop externally, techniques such as microinjection or electroporation allow for simpler introduction of transgenes than in mammals, which usually require transfection. To this end, a number of transgenic models of heart damage have been developed in the lower vertebrates. The inducible nature of these models, usually by exposure to or injection of a drug, prevents the transgene from interfering with normal heart development and allows for the creation of a disease phenotype within a highly specific time period.

The first such tool to be developed for use in a heart regeneration study was the Nitroreductase (NTR)/Metronidazole(Mtz) system, a genetically expressed, inducible, targetable method of cell ablation. *Escherichia coli* nitroreductase is expressed in the target organism, usually under a tissue- or cell type-specific promoter. Upon introduction of the prodrug Metronidazole [1-(2-hydroxyethyl)-2-methyl-5-nitroimidazole], a substrate of NTR, the Mtz is reduced to a potent DNA cross-linking agent. Death of the host cell via apoptosis follows within hours to days. The toxic form of Mtz does not cross cell boundaries, making this method highly cell-specific [38,39].

The NTR/Mtz system has so far been used for heart studies only in larval zebrafish. The NTR gene has been expressed under the *cardiac myosin light chain 2* (*cmlc2*) and *ventricular myosin heavy chain* (*vmhc*) promoters, allowing for either heart-wide expression or expression limited to specific regions of the heart. NTR can be conjugated to fluorescent proteins, including CFP and mCherry, allowing for visual identification of transgenic organisms and tissues. The zebrafish heart regenerates after Mtz-induced cell ablation, and since Mtz can be washed out, the regenerated cardiomyocytes will not themselves undergo further apoptosis [38,40].

Another tool that has been used for inducible, targeted cell ablation in zebrafish is the Z-CAT (zebrafish cardiac ablation transgenes) system, which uses two transgenes to restrict cell death to a target population. The first construct contains a fusion of Cre recombinase and estrogen receptor (CreER) to create a Cre that is inducible by 4-hydroxytamoxifen (4-HT). In order to restrict expression to cardiomyocytes, it is placed under a *cmlc2* promoter. The second transgene is the *bactin2:loxp-mCherry-STOP-loxp-DTA* construct; the *β-actin2* promoter further restricts activity to the myocardium, where, upon 4-HT induction, diphtheria toxin A-chain (DTA) is produced. This approach induces apoptosis in the target cells within 2 days of 4-HT exposure [41]. A total of ~60% of cardiomyocytes are ablated throughout the course of this process, resulting in severe heart failure phenotypes. Unlike NTR/Mtz, this method is not reversible; however, regeneration still follows within 1–3 weeks after 4-HT exposure [41,42,43].

A major advantage of genetic approaches is that they do not require any invasive surgery or manipulation, reducing the likelihood of off-target effects. The choice of promoter in the transgenic animal restricts expression of the transgene—and therefore cell death—to a specific cell type, which is both advantageous and disadvantageous. On the one hand, the ablation target is highly specific, allowing a researcher to study cardiomyocyte regeneration in isolation from the other layers of the heart. However, natural diseases like MI may result in the death of multiple cell types within a region, something these methods fail to replicate [29]. Regeneration from genetic ablation also takes place within a greatly accelerated timeframe compared to cryoinjury or resection, occurring within two weeks in the Z-CAT model [37]. The differences between repair of a contiguous region of missing (or necrotic) tissue, and repair of scattered ablation of cells, may therefore limit how applicable these results are to repair of infarcts. However, necrosis and apoptosis akin to the effects of genetic ablation do play a role in several other types of heart disease and heart failure, which these techniques can help to study. Ischemia, oxidative stress, alkalosis, and other conditions not caused by physical trauma to the heart can lead to an increase in both apoptosis and necrosis throughout the myocardium [44]. Furthermore, cell death is a causative factor in heart failure and associated conditions, and can cause mortality in as few as 0.08% of cardiomyocytes. Understanding how organisms regenerate from dispersed cell death can therefore be a highly useful tool in treating many forms of heart failure [45,46]. Overall, the choice of genetic or mechanical methods of ablation depends greatly on what sort of injury and repair process the researcher wishes to investigate.

### 2.4. Environmental Conditions

A number of cardiac diseases in humans are caused by either the presence of damaging agents in blood, such as reactive oxygen species, or deprivation of essential resources, such as oxygen. Oxidative stress, hypoxia [47], acidosis [48], and osmotic stress [49] are among the conditions with an adverse effect on cardiac function that result from a change in blood composition.

A major advantage of aquatic model organisms is the ability to replicate these conditions by simply changing the components of the solution that the animal is living in, altering pH, oxygen levels, or presence of cardiotoxins. For fish or amphibian larval stages, diffusion is usually sufficient to expose the heart to the solute of interest [50]. However, adult amphibian stages, especially of those species living on land, may require removal of the heart to perfuse it with solution [51]. In this manner, the gas content, pH, and presence of any solutes in the lumen of the heart can be precisely controlled. The disadvantage of this technique is that the heart can only be looked at in isolation, rather than as part of a functioning whole organism.

Much like genetic ablation models, such treatments cause dispersed cell damage or death, and as such are not appropriate for mimicking infarction or other acute forms of trauma. Because most environmental conditions do not induce as drastic a damage phenotype as mechanical methods like amputation or cryoinjury, these techniques are more commonly used for the study of non-regenerative heart repair. A series of studies on the ability of the frog *Rana ridibunda* to protect against heart damage has examined the effects of many diverse environmental factors, including: hyperosmosis, via increased NaCl and KCl concentration [52]; oxidative stress, via hydrogen peroxide [53]; hypoxia, either via replacement of oxygen in solution with nitrogen [51], or by addition of cobalt (II) chloride [54]; acidosis and alkalosis via buffers of the respective pH [55]; and mechanical overload, via increased perfusion pressure [56]. Environmental conditions have also been used in the study of heart repair in fish. By exposing zebrafish to hypoxic conditions, and then returning them to oxygenated water, it is possible to replicate the oxidative stress that occurs following ischemia/reperfusion in human hearts [50].

## 3. Heart Regeneration in Fish and Amphibians

While very different in gross morphology, the hearts of fish and amphibians have much in common with each other and with mammalian hearts. The vertebrate heart consists of three main layers—the endocardium, an endothelial layer lining the inner lumen of the heart; the myocardium, the muscle layer; and the epicardium, a protective sheet of epithelial cells covering the outside of the myocardium. The myocardium is itself composed of two layers: the compact myocardium that forms the walls of the heart, and the trabecular myocardium, which forms the contractile trabeculae on the interior of the heart chambers [21,24]. Additionally, the general pattern of heart development is well conserved among the vertebrates: a symmetrical pair of *nkx2.5*-positive mesodermal regions, known collectively as the heart field, help to establish the cardiac precursor cell population. During early development, these fields migrate to the midline of the organism and converge to form a linear heart tube. This tube then loops into an S-shape, as the cells along its axis differentiate into atrial and ventricular cells. Extracardiac populations of cells meanwhile migrate to the heart to form the endocardium and epicardium. In fish, no further chamber septation occurs; in amphibians, the atrium separates in two. Both taxa have only a single ventricle, unlike mammals [57].

Befitting such similarities in development and morphology, heart regeneration follows a highly stereotyped behavior, independent of species or most methods of damage. Within the first hours to days after injury, clotting begins to seal the wound, followed by an inflammatory response that leads to deposition of a collagen and fibrin extracellular matrix (ECM) by epicardium-derived fibroblasts. Next, cardiomyocytes outside the injury zone partially dedifferentiate to reenter the cell cycle, proliferate, and migrate to the wound site. The fibrotic material filling the wound is replaced over the following days to weeks with functional cardiomyocytes (summarized in Table 1). Depending on the exact damage model studied, additional steps may be required. For example, cryoinjury, which replicates the effects of an infarct, results in necrotic tissue that needs to be cleared before fibrosis and regeneration can continue [35]. Conversely, certain methods of damage may not require some of these steps during repair. Z-CAT genetic ablation, for instance, results in minimal collagen deposition [41].

**Table 1:** Timeline of main events in cardiac regeneration by organism and damage model. Times listed are the earliest point at which the process was observed (d = days post injury). A dash (—) indicates that no data exists for this model.

	Inflam-mation	Epicardial Activation	Fibrosis		Cardiomyocytes			Recovery (Function)	Recovery (Morph.)
			Fibrin	Collagen	Dediff.	Prolif.	Migration		
***D. rerio* (adult)**									
*Amputation*	<1 d [58]	1 d [59]	2 d [21]	14 d [21]	7 d [60]	7 d [21]	9 d [21]	30 d [61]	60 d [21]
*Cryoinjury*	1 d [35]	3 d [35]	4 d [36]	7 d [36]	4 d [62]	3 d [35]	14 d [37]	30 d [36]	60 d [36]
*Ablation (Z-CAT)*	3 d [41]	7 d [41]	—	14 d [41]	7 d [41]	7 d [41]	—	14 d [41]	30 d [41]
***D. rerio* (larva)**									
*Ablation (NTR)*	—	1 d [40]	—	—	1 d [40]	2 d [40]	2 d [40]	4 d [38]	4 d [38]
***D. aequippinatus***									
*Cautery*	1 d [22]	—	—	7 d [22]	—	7 d [22]	14 d [22]	—	45 d [22]
***C. auratus***									
*Cautery*	3 d [23]	—	7 d [23]	14 d [23]	14 d [23]	3 d [23]	—	—	45 d [23]
***N. viridescens***									
*Amputation*	5 d [17]	—	7 d [26]	7 d [26]	—	7 d [26]	30 d [14]	23 d [26]	60 d [26]
*Crushing*	—	—	—	1 d [63]	1 d [27]	—	—	14 d [27]	84d [63]

The major processes involved in heart regeneration—epicardial activation, fibrosis, and cardiomyocyte processes, including dedifferentiation, proliferation, and migration—are described in greater detail below. Most of the specific results were reported in zebrafish, although amphibian experiments have shown that many of these processes are conserved across vertebrate taxa. Where the regeneration process differs in other organisms, it is specifically noted.

### 3.1. Epicardial Activation

A key component of heart regeneration in numerous species and models of damage is the role of the epicardium. The epicardium and the tissue types derived from it play an important part in cardiac development, where epicardium-derived cells (EPDCs) differentiate into numerous cell types, including coronary vascular smooth muscle and fibroblasts [64]. During heart regeneration, epicardial processes include reactivation of developmental genes in epicardial cells and the transdifferentiation of epicardiocytes into other cell types necessary for regeneration to proceed, including fibroblasts. Epicardial activation begins with re-expression of *wt1*, a marker of stem cells and developing epicardium in particular, as well as other epicardial developmental genes like *raldh2* (necessary for retinoic acid synthesis) and *tbx18*, all within the first 1–2 days after injury. *Raldh2* is most active during early regeneration; it is first expressed in the atrial and ventricular epicardium days after the injury, is localized to the site of the injury by one week, and diminishes in expression by two weeks after injury. Meanwhile, *tbx18* expression persists much longer, at least one month post-injury [24,35,59,65,66].

Regeneration of the epicardium itself occurs relatively early in the regeneration process, occurring before restoration of the myocardium is visible [22]. Activated epicardial cells begin to proliferate approximately three days after injury, and by two weeks, the epicardium has enclosed the injured area, providing protection and support for regeneration of the myocardium [59,67].

Approximately one week after injury, epicardial cells upregulate expression of *snail2* and *twist1b*, markers of the epithelial-to-mesenchymal transition (EMT), in preparation for transdifferentiation [68]. Beginning at 2 weeks post-injury, activated epicardial cells then infiltrate the myocardium, where they downregulate *wt1* expression and differentiate into a variety of EPDC cell types [65]. Some migrate into the regenerating coronary vasculature to become vascular pericytes and organize neovascularization, a process mediated by platelet-derived growth factor (PDGF) and fibroblast growth factor (FGF) signaling [59,65,68,69]. Others express fibronectin and transdifferentiate to fibroblasts, which infiltrate the injury site to first deposit collagen, and later promote migration of cardiomyocytes into the wound to replace fibrotic tissue [42]. One important cell type not found in this list is cardiomyocytes; epicardial cells that undergo EMT do not subsequently express myocardial differentiation markers like myosin heavy chain (MHC) or *cmlc2*. Similarly, they do not express the endothelial marker *fli1*, indicating that endocardium is also not among the potential fates of EPDCs [65,69].

### 3.2. Cardiomyocyte Dedifferentiation and Proliferation

In order for injured or infarcted myocardium to regenerate, new cardiomyocytes must be produced; where, then, do they come from? Early studies in zebrafish heart regeneration indicated that the cardiomyocytes involved in regenerating missing myocardium were formed through differentiation of an undifferentiated progenitor cell population [59]. However, subsequent experiments have shown that all regenerated cardiomyocytes originate from existing cardiomyocytes [60,61]. Since adult cardiomyocytes are largely quiescent, regeneration requires them to reenter the cell cycle, which is controlled in part by the mitotic checkpoint kinases Mps1 and Plk1. Inhibition of either of these kinases prevents myocardium from replacing fibrotic tissue and leads to permanent scarring [21,60]. Also necessary for proliferation is the gap junction protein Cx43. Mps1 and Cx43, along with numerous other cell-cycle regulators, structural proteins, and cardiomyopathy-associated genes are regulated by the miRNA miR-133, downregulation of which is essential to create a permissive environment for cardiomyocyte proliferation to proceed [70].

Reentry of cardiomyocytes into the cell cycle also requires dedifferentiation and re-expression of several cardiac development genes [59,61]. The question of which genes are reactivated, and exactly to what extent dedifferentiation occurs, is unclear. Some groups have reported expression of the early developmental genes *nkx2.5*, *hand2*, *tbx5*, *tbx20*, and *mef2* in regenerating hearts [40,59], while others have observed no expression of these genes [28,60]. *Gata4*, an early cardiac development gene and one of the principle markers of this dedifferentiation process, has in contrast repeatedly been observed in both fish and amphibians [26,61,71]. *Gata4* expression is re-induced in cells of the outer compact myocardium, predominantly near the injury site, and these *gata4^+^* cells are what proliferate and fill the wound site [61]. Also upregulated in the regenerating myocardium is the homeobox gene *msx*, a gene that regulates regeneration across species and organs, including newt limbs and zebrafish fins. However, *msx* is not upregulated in heart development, indicating that the regeneration process is more complex than just a recapitulation of cardiac development [28].

Morphologically, dedifferentiation is visible in both fish and amphibian cardiomyocytes in the form of disorganized sarcomeres, loss of intercellular cohesion, a rounding of cell shape, and downregulation of terminal differentiation cardiac proteins like myosin and troponin [27,40,41,59,60,72]. Fewer experiments have looked at gene expression in amphibians during heart regeneration, but one of those that has showed an increase in *nkx2.5*, *hand2*, *gata4*, *gata5*, and *islet1* [26], the latter of which has not been observed in fish heart regeneration [40].

This combination of proliferation and dedifferentiation is indicative of epimorphic regeneration, in which an undifferentiated blastema forms at the site of injury, and undergoes differentiation to replace the missing or infarcted tissue. In zebrafish, this is manifested as an accumulation of highly proliferative blastemal cells expressing embryonic markers in the immediate vicinity of the infarct zone, beginning at 4 days post-injury. In addition to this immediate local response, there is also an increase in proliferation throughout the rest of the uninjured heart that persists to later stages of regeneration. The distribution of mitotic cells shifts away from the infarcted region as regeneration progresses, possibly indicating that later phases of regeneration are associated with heart-wide systemic repair [58]. This late, ventricular-wide increase in proliferation has also been observed in the newt heart during regeneration [67].

One particularly interesting source of cardiomyocytes during ventricular repair, observed in both amphibians and fish, is the atrium. Atrial myocardial proliferation in response to ventricular damage was first observed in the newt, *Notophthalmus viridescens*, where in fact proliferation was more prevalent in the atrium than in the ventricle [16]. Another amphibian species, the axolotl *Amblystoma mexicanum*, showed a comparable increase in atrial mitosis during regeneration of the ventricle [20].

In zebrafish, an atrial response is visible even before an increase in proliferation occurs. *Raldh2* is upregulated in the atrial endocardium within an hour after ventricular injury, hours before it is upregulated in the ventricle [24]. At one day post-injury, activation of the uninjured atrial epicardium begins simultaneously with that of the injured ventricle, with *raldh2* expression followed by *tbx18* upregulation. Expression of these markers, however, soon restricts itself to the area immediately adjacent to the ventricular wound. Meanwhile, fibroblast growth factor receptor (*fgfr*), associated with cardiomyocyte migration into the wound, is activated in contiguous patches throughout the atrial epicardium [59]. These findings suggest that activation of all three layers of the atrium is involved in cardiac regeneration.

The role of atrial cardiomyocytes in ventricular regeneration has been confirmed in zebrafish using a cell ablation model. When ventricular cardiomyocytes are ablated, atrial cardiomyocytes dedifferentiate into cardiac progenitor cells. These cells show disorganized structure, decreased expression of terminal differentiation markers like MHC, and re-expression of developmental genes including *gata4*, *hand2*, *nkx2.5*, *tbx5a*, *tbx20*, and *mef2*. The progenitor cells then reacquire a ventricular identity as they migrate into the ventricle to become mature ventricular cardiomyocytes. The migration process is dependent on Notch signaling, and upregulation of both Notch pathway components and *raldh2* occurs in the atrial endocardium, indicating a possible role for retinoic acid (RA) as well [40].

In addition to its role in epicardial activation, RA is necessary for cardiomyocyte proliferation. *Raldh2* is activated by the inflammatory response in the atrial and ventricular endocardium within hours after injury, and within a day becomes localized to the site of the injury. These *raldh2*-positive cells are also enriched for expression of cardiac developmental genes *hand2* and *gata5*. Along with *raldh2*-positive epicardial cells, the endocardium makes up one of the two major sources of RA during heart regeneration. Inhibition of RA signaling from endocardial and epicardial cells greatly reduces injury-induced cardiomyocyte proliferation. However, overexpression of RA has no effect on proliferation levels, indicating that RA signaling is necessary, but not sufficient for proper cardiomyocyte proliferation [24]. The endocardium is also responsible for producing Interleukin 11a (Il11a), a ligand of the Jak1/Stat3 signaling system. Jak1/Stat3, expressed in the myocardium, and its downstream effectors are likely involved in cell survival during the early, wound-healing portion of regeneration, and are required for later cardiomyocyte proliferation [73].

A number of other paracrine and juxtacrine signaling pathways, involving all three layers of the heart, are also necessary for cardiomyocyte proliferation. These include PDGF [74], Hedgehog, Insulin-like growth factor (IGF), TGF-β, and Notch [43]. Sonic hedgehog (Shh) is activated in the injury-adjacent epicardium, with downstream markers of Shh activity observed in adjacent cardiomyocytes. IGF is expressed in the endocardium, with its receptor present in the injured myocardium [43]. TGF-β is present in the myocardium, epicardium, and fibroblasts [37]. The Notch receptor, meanwhile, is upregulated in both the endocardium and epicardium, but remains absent in the intervening myocardium [71]. The expression pattern of Notch ligands is less well-studied; only *deltaC*, expressed in the endocardium alongside the *notch1b* isoform, has been examined. Notch receptor is expressed in the heart from 1 day to 1 week after injury, after which the gene is downregulated [28]. Notch signaling is necessary for proliferation of cardiomyocytes; when it is inhibited during the later period of Notch expression (6–7 days post-injury), the wound remains fibrotic and regeneration of the myocardium never takes place. Interestingly, hyperactivation of Notch signaling also prevents cardiomyocyte proliferation, indicating that a very specific level of signaling is necessary for regeneration to proceed properly [71].

Several *in vitro* experiments on cultured newt cardiomyocytes have further elaborated on the factors necessary for myocyte cell cycle reentry and proliferation. The major restriction point in beginning this process is the retinoblastoma protein (Rb), which in wild-type tissues prevents cells from entering S phase. Phosphorylation of Rb via cyclin-dependent kinase (Cdk) 4 and 6 deactivates this S-phase restriction and is necessary for newt cardiomyocytes to enter the cell cycle [75,76]. However, entry into S phase does not ensure subsequent proliferation. While most cardiomyocytes are able to enter S phase in culture, only one-third of those subsequently complete one or more rounds of cell division, with the majority arrested just before mitosis or before cytokinesis [76]. This separation between cell cycle reentry and proliferation is borne out by the fact that while PDGF and IGF promote cardiomyocyte proliferation, they cannot by themselves induce terminally differentiated myocytes to reenter the cell cycle [75]. Multiple ECM components have also been demonstrated to induce proliferation in cardiomyocytes in culture, including laminin, fibronectin [76], and tenascin C [67].

Proliferation, however, is not enough by itself; after cardiomyocytes proliferate, they must then be induced to migrate into the wound region. Inextricably bound to this process of cardiomyocyte migration is fibrosis, a crucial step in cardiac regeneration that bridges the gap between early wound healing and later regeneration of myocardium.

### 3.3. Fibrosis and Cardiomyocyte Migration

Among the first events required in cardiac regeneration, as part of a general wound-healing response, are clotting, activation of the inflammatory response, and fibrosis. In resection and cryoinjury models, these events occur primarily within the first week after injury. Clotting begins within seconds of injury, as the wound is sealed by erythrocytes [21]. Within the first day, activated thrombocytes enter the wound, and over the following weeks express PDGF to aid in neovascularization, and other factors necessary for regeneration [33,68]. Beginning at 3 h post-injury, and continuing over the next several days, the inflammatory response peaks as leukocytes, including macrophages and myeloperoxidase-positive neutrophils, begin to infiltrate the wound site [22,35,41,62,74]. Inhibition of the inflammatory response leads to a decrease in recruitment of phagocytes, impairment of angiogenesis, and decrease in cardiomyocyte proliferation, and ultimately prevents regeneration from completing [62].

At the same time as inflammation, apoptosis and/or necrosis begins within the injury area (or injury-adjacent area in resection experiments), as the cellular debris in the infarct is cleared away [33,35,36,63]. Simultaneous with inflammation and initiation of apoptosis, the erythrocyte clot is replaced by a temporary fibrin matrix, which retains structure in the wound until approximately one week post-injury, when the temporary matrix is lysed to be replaced by a primarily collagen matrix and initiate the phase of regeneration dominated by fibrosis [21,36].

Fibrosis, or scarring, is a process of wound repair that involves replacement of missing or necrotic tissue with connective tissue, primarily fibrin and collagen. In adult mammalian hearts, this is the only process by which lesions or infarcts can be repaired, but permanent scarring leads to changes in heart contractility, conductivity, and morphology that can greatly reduce the function of the heart [33]. In organisms that can regenerate their hearts, this fibrotic tissue is soon replaced by functional myocardium and no permanent scar persists. While scarring and regeneration are commonly seen as opposing outcomes, an initial period of fibrosis is necessary for proper regeneration in both fish and amphibians, providing a framework in the form of ECM for cardiomyocytes to assemble into working muscle [37,63,67]. During the period of fibrosis, a number of wound healing genes, such as *vegf* to promote vascularization and *granulin A* to promote cell growth, are upregulated [74]. In order for regeneration to be completed, newly proliferated cardiomyocytes need to be able to migrate into the injured region, and simultaneously, the fibrotic tissue needs to be removed. Beginning at one week, therefore, remodeling proteins such as matrix metalloproteinases (MMPs) are activated, and resolution of fibrosis begins [74].

The TGF-β signaling pathway is an essential component of both establishing and resolving fibrosis. During regeneration of the zebrafish heart, within the first two weeks after injury, TGF-β is expressed in the myocardium, epicardium, and fibroblasts of the injured region and infarct boundary, but is not found in the endocardium. [37,41] Multiple isoforms of the TGF-β receptor are also present, and the infarct zone and adjacent tissue show markers of TGF-β activity in the form of phosphorylated Smad3 [37].

TGF-β is required in both early and late phases of heart regeneration. Within the first two weeks, it is predominantly necessary for inducing fibroblasts to deposit fibronectin and collagen into the injury site, forming the ECM that provides a framework for later muscle regeneration. Inhibition of TGF-β signaling after two weeks does not affect the collagen matrix, but it does prevent cardiomyocytes from infiltrating and replacing the fibrotic ECM. TGF-β regulates expression of the glycoprotein tenascin C at the infarct boundary, which in turn is necessary for remodeling of the ECM and migration of cardiomyocytes into the wound region. [37] Fibronectin may also play a role in this migration process, as it appears to be required for signaling integrin receptor-expressing cardiomyocytes, and inhibition of it prevents regeneration [42]. This role for the ECM in guiding regeneration is conserved in amphibians, where fibronectin, tenascin C, and hyaluronic acid are upregulated in the wound region throughout regeneration, and in the case of the latter two up to 70 days post-injury [67]. In fish, the migrating cardiomyocytes are primarily directed by the chemokine Cxcl12-Cxcr4 system [66], and build new myocardium in the injured area from the outside of the wound (apical edge of the myocardium) inward [33,59]. New cardiomyocytes begin to become electrically coupled around 14 days post-injury, and are fully coupled by 30 days [36,61].

FGF signaling is also necessary for proper regeneration and resolution of scarring. FGF is secreted by myocardial cells adjacent to the injury site. This process activates FGF receptor-expressing epicardial cells, and is necessary for the collagen scar to be replaced by myocardium; while the mechanism for this signaling process has not been characterized, the similarities in effect to TGF-β inhibition suggest that FGF’s role in epicardial activation may follow a similar pathway [59]. Interestingly, if FGF signaling is inhibited to prevent regeneration and induce scarring, the scarring process can be partially reversed by later restoration of FGF. While fibrotic tissue is never fully resorbed, myocardium is restored over the scar. This may indicate that scarring is a non-permanent outcome, and may provide an avenue for repairing existing infarct damage in patients [61].

## 4. Non-Regenerative Heart Repair

In addition to the prodigious regenerative capabilities possessed by certain species, many fish and amphibians are capable of repairing lower levels of heart damage that may not require a full regenerative response. The organs of ectothermic animals, including the heart, are more subject to changing environmental conditions than those of endothermic animals. As a result, they have developed adaptations that allow them to survive a wider range of temperatures, gas concentrations, and pH values. Some of these adaptations are protective, active before damaging agents are able to adversely affect cardiac function; these include robust antioxidant systems, ion pumps to prevent extreme pH values, and ability to maintain cardiac function during hypoxia [77]. However, those particular adaptations lie outside the scope of this review; for more information, see Driedzic *et al.* [77] Instead, we will focus on mechanisms associated with post-damage compensation and repair.

Since urodele amphibians and certain fish species are so adept at regenerating, most studies in these organisms have focused on full regeneration. As a result, they have not been used as often to study repair in a non-regenerative context, although some research has been conducted on the ability of zebrafish to repair after reperfusion injury [50]. Instead, lower vertebrate species that are not capable of regeneration have been used as the primary model for this type of repair, no group of animals more so than the anuran amphibians. While full cardiac regeneration has not been observed in anurans to date, they have proven to be a useful model for studying amphibian tolerance to low levels of damage, particularly oxidative and mechanical stress. A primary regulator of the stress response in amphibian, as well as mammalian, hearts is the mitogen-activated protein kinase (MAPK) family. MAPKs are activated by a variety of environmental and cytokine stimuli, and in turn phosphorylate important cardiac transcription factors (e.g., Mef2) and general cell regulation factors (e.g., c-Jun). These ultimately regulate a wide range of cell behaviors from apoptosis to cell survival and cardiomyocyte hypertrophy [78].

In the amphibian heart (mainly studied in the adult marsh frog, *Rana ridibunda*), MAPK activity is induced by stress stimuli including hyperosmosis, anoxia, and reperfusion [51]. One subset of MAPKs in particular, the p38-MAPK family, has been shown to be activated in frog hearts by a number of common disease-associated stimuli, including hypoxia [54], osmotic stress [52], reactive oxygen species [53,79], acidosis [55], and mechanical overload [56]. This p38-MAPK activity in turn confers protective effects on cardiomyocytes via phosphorylation of MAPK-activated protein kinase 2 (MAPKAPK2) and Heat shock protein 27 (Hsp27), both associated with cell survival [54,55,79]. One potentially important downstream target of this pathway is atrial naturetic peptide (ANP), a hormone responsible for regulating ion and fluid balance across the cell membrane, and commonly associated with stress compensation. ANP levels have been shown to increase in the frog heart after hypoxic or osmotic stress [52,54]. Additionally, changes in extracellular pH induce expression of the chaperone protein Hsp70, although this occurs independently of p38-MAPK activity [55]. Other classes of MAPKs, specifically the c-Jun N-terminal kinases (JNKs) and extracellularly responsive kinases (ERKs), have also been shown to be activated by stressors in the frog heart [51,53,56]. Overall, the MAPK-mediated stress response appears to be a crucial component of protecting and repairing low-level, environmental damage to the anuran heart.

What happens, however, if an anuran heart is damaged to an extent that would induce regeneration in a urodele or fish heart? Years before the regenerative capacity of newts was discovered, Rumyantsev observed that the adult common frog (*R. temporaria*) heart could partially repair damage induced by crushing the ventricle [80,81]. Within the first week after damage, phagocytes enter the wound region to remove necrotic myocardium and transcription in neighboring myocytes increases. In the second and third week, myocytes partially dedifferentiate and re-enter the cell cycle. Myofibers then extend into the wounded region, connecting the muscle on either side of the lesion. Unlike in regeneration, however, myocardium is never fully restored across the lesion, and scar tissue remains [81].

The question of why anurans and urodeles differ in regenerative capacity is still not well understood. Observation of mitotic kinetics in frog heart repair has shown that myocytes proliferate twice as slowly as fibrotic tissue [81]; however, post-damage myocyte proliferation is comparable between non-regenerating frogs and regenerating urodeles, indicating that this is likely not the main driving force [20,81]. Embryonic anurans are capable of regenerating many structures that do not regenerate in adult anurans, but do regenerate in adult urodeles, including limbs [82], the lens of the eye [83], and the brain [84]. It is likely that by understanding the differences in regenerative capacity between these three systems—embryonic anurans, adult anurans, and adult urodeles—we can better determine how to induce cardiac regeneration in non-regenerative organisms.

## 5. Conclusions

While much of the research on heart damage and repair has understandably been conducted in mammalian models to date, fish and amphibians provide a unique opportunity for studying a system that can completely regenerate missing or damaged cardiac tissue. Regeneration in mammalian hearts is limited-to-nonexistent, and scarring—with a resulting loss of heart function—is the most common result of cardiac damage. What little evidence of cardiac renewal exists occurs mostly in neonatal or embryonic mammals, though adult mammal hearts are not entirely devoid of restorative processes. Myocyte proliferation has been observed in response to human heart damage [7], *raldh2* activation occurs in the endocardium of infarcted mouse hearts [24], and epicardial activation and cardiomyocyte dedifferentiation, hallmarks of the regenerative response in zebrafish, occur after resection of neonatal mouse hearts [11]. In contrast, throughout their lifetimes, some species of fish and urodele amphibians can restore all three layers of the heart with no permanent scarring and full restoration of functionality.

In addition to the improved regenerative response of fish and amphibians, there exists a plethora of physical and molecular tools for studying heart regeneration in these model systems. Mechanical manipulations and other physical techniques like cryoinjury are significantly easier in fish and amphibians than mammals, particularly at embryonic stages. The wealth of genomic data for zebrafish also makes transgenic methods of damage, which can be used to minimize off-target effects and unnecessary surgery, an extremely viable solution. And while fish are the more ubiquitous model, amphibians should not be forgotten; the amphibian heart is more similar to the mammalian heart, molecularly and morphologically, than that of fish. Differences in regenerative capacity between urodele and anuran amphibians may also prove useful in understanding why some organisms can regenerate tissue while others, like mammals, use scarring as their main method of repair.

With a greater understanding of heart regeneration in amphibians and fish, and the effect that has had on understanding mammalian heart repair, potential new therapeutic strategies for mammals are being devised. One major obstacle is the relative lack of proliferation in mature mammalian cardiomyocytes, and so inducing them to reenter the cell cycle and proliferate has been a large focus of research. Targets that have shown success on this front, many of which have been observed to play a role in lower vertebrate heart regeneration and repair, include FGF, p38-MAPK [85], periostin [86], neuregulin [87], and cyclins and CDKs [88]. The level of conservation between these processes in lower vertebrates and mammals has been demonstrated by the ability of newt regeneration extract to induce dedifferentiation of mammalian myocytes [89]. MicroRNAs, which in addition to being involved in many cardiac remodeling processes have proven to be necessary for cardiomyocyte proliferation in zebrafish heart regeneration [70], have also been a promising target for inducing cardiac repair through regulation of endogenous gene expression [90]. The other major focus in inducing mammalian heart repair, besides myocyte proliferation, has been the role of EPDCs. While EPDCs do not directly lead to new myocardium, they promote a permissive environment through secretion of ECM by fibroblasts and promotion of angiogenesis. Improving the pro-regenerative capacity of mammalian EPDCs, while limiting fibrosis from the same, may prove to be as important a step towards inducing regeneration in the mammal heart as making new cardiomyocytes [91,92].

The regenerative abilities of fish and amphibians are just beginning to be understood. They have already proven to be a valuable model system for researching cardiac damage and repair, and their importance should only increase going into the future. By understanding how regeneration is possible in fish and amphibians—and why mammalian hearts fail to regenerate, despite the presence of conserved repair responses and pathways—we can hopefully discover novel ways to stimulate repair in mammalian hearts that can serve as therapeutics in human heart disease.
